# Facial pyoderma gangrenosum associated with fallopian tube carcinosarcoma

**DOI:** 10.1002/ccr3.8065

**Published:** 2023-10-15

**Authors:** Ghazal Mardani, Mohammad Shahidi Dadras, Fahimeh Abdollahimajd, Toktam Safari Giv, Elnaz Pourgholi, Azadeh Rakhshan, Fariba Ghalamkarpour

**Affiliations:** ^1^ Skin Research Center Shahid Beheshti University of Medical Sciences Tehran Iran; ^2^ Pathology Department Shahid Beheshti University of Medical Sciences Tehran Iran

**Keywords:** carcinosarcoma, malignancy, pyoderma gangrenosum, ulcer

## Abstract

Pyoderma gangrenosum (PG) is a neutrophilic dermatosis associated with underlying disorders. The association between PG and solid organ tumors (SM), including gynecologic cancers, has been previously reported. Here, we report a case of a 61‐year‐old woman with pyoderma gangrenosum on the posterior auricular region associated with an underlying fallopian tube carcinosarcoma: a rare and aggressive gynecologic malignancy. The patient's ulcer responded favorably to treatment, and surgical resection of the tumor was performed. The patient was then referred for further cancer management. No new lesions or recurrences were found over the 18 months of routine follow‐up.

## Graphical abstract

Pyoderma gangrenosum was associated with an underlying fallopian tube carcinosarcoma: a rare and aggressive gynecologic malignancy in this patient.

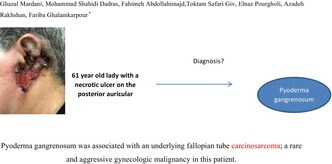

## 1 INTRODUCTION

Pyoderma gangrenosum (PG) is a rare noninfectious neutrophilic dermatosis characterized by painful ulcers with violaceous borders. PG usually affects the lower extremities, although it can involve other parts of the body including the mucous membranes. However, involvement of the face is rare.^1^

The diagnosis of PG is based on ruling out other similar conditions.^2^ This disease is classified into four types: bullous, ulcerative, pustular, and superficial granulomatous.^3^ In 2004, Su et al.^4^ proposed a diagnostic criterion for PG including major and minor criteria. Here, we present an unusual case of PG in terms of location and the associated malignancy.

## 2 CASE PRESENTATION

**FIGURE 1:**
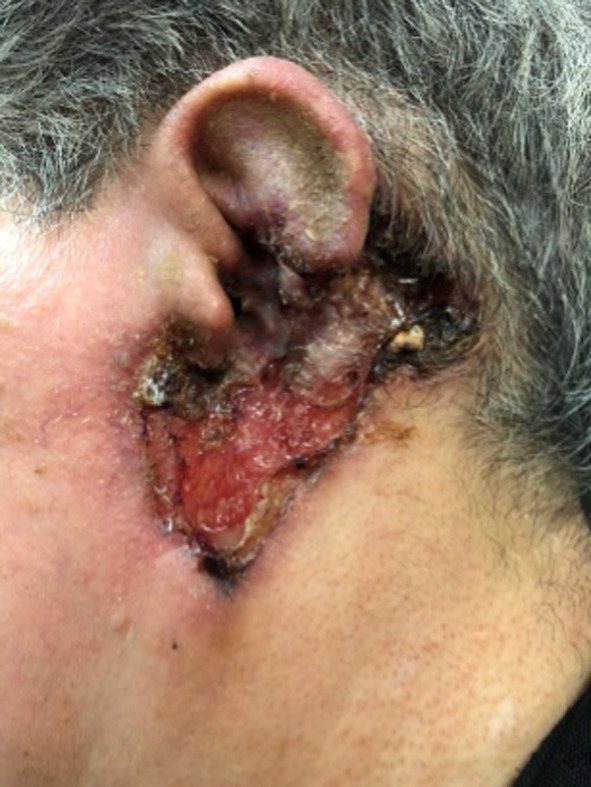
Necrotic ulcer, with irregular, violaceous, borders affecting the lower third of the earlobe.

A 61‐year‐old woman presented with a painful necrotic ulcer, located on the left periauricular region starting from the lower border of the tragus, extending toward the mandibular angle, affecting the lower part of the ear and posterior border of the ear of one‐year duration. (Figure 1). She had no significant drug or social history, except for unilateral oophorectomy and hysterectomy due to abnormal uterine bleeding (AUB) 15 years ago. Despite multiple biopsies and diagnostic work‐ups for the facial lesion, no specific diagnosis had been established. The biopsy reports had been nonspecific. Various tests, including evaluations for deep mycosis, acid‐fast bacilli, atypical mycobacteria cultures, PAS, ZN stain, and leishman body smear, all returned negative. The patient had also received multiple courses of antibiotics without any significant response. Consequently, ENT specialists decided to debride the necrotic tissue, which unfortunately exacerbated the ulcer's severity.

**FIGURE 2:**
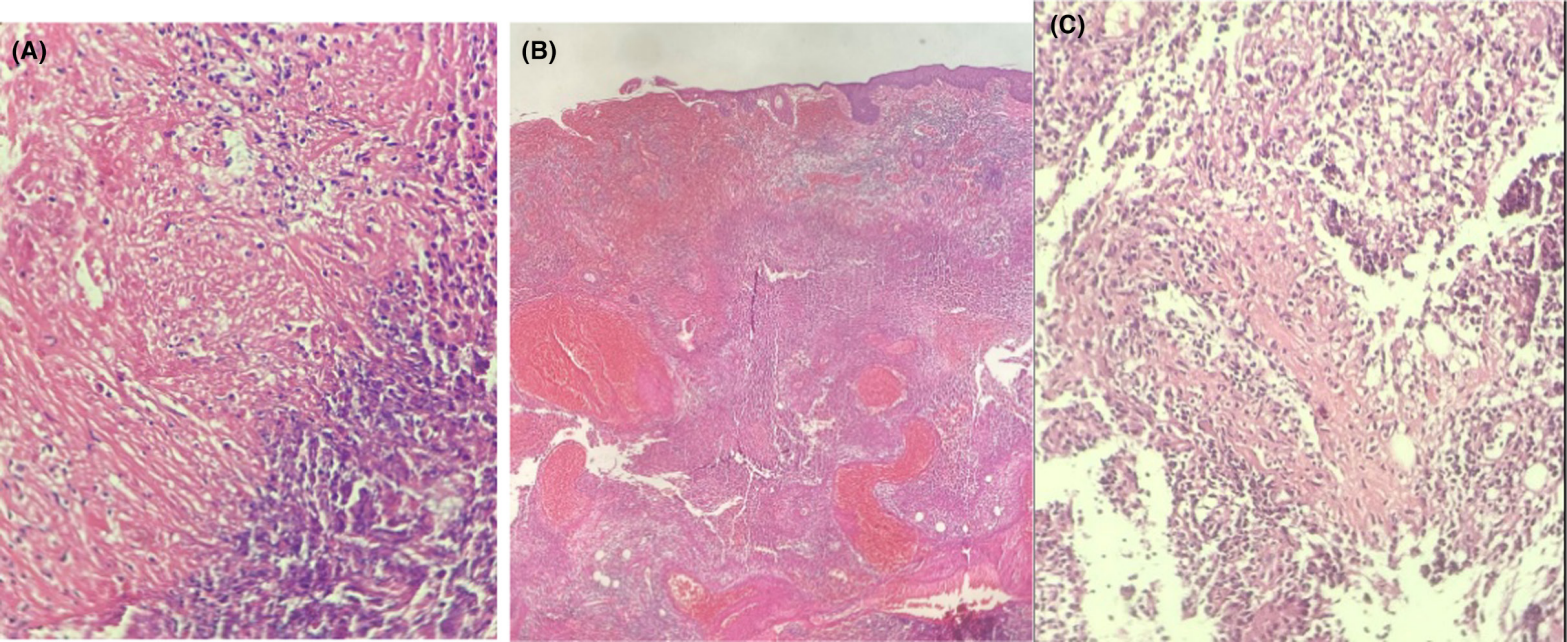
Histopathology results revealed, dermal necrosis (A), necrosis and acute inflammation (B), and infiltration of vascular wall and vasculopathy(C).

The patient was referred to our clinic with a large, deep, and tender necrotic ulcer with a raised border and destruction of the lower part of the ear extending toward the scalp and mastoid area. She was admitted for further evaluation, considering PG and granulomatosis with polyangiitis (GPA) in the differential diagnosis. A comprehensive systemic analysis was carried out, including smear and culture from the ulcer, complete blood count, urine analyses, liver and renal function test, serology tests for hepatitis B and C as well as human immunodeficiency virus antibody test, antinuclear antibodies, cytoplasmic and perinuclear and neutrophilic cytoplasmic antibodies (c‐ANCA and p‐ANCA), serum electrophoresis, chest, paranasal sinuses, and mandibular X‐ray. The only positive result was the growth of Klebsiella in the bacterial culture from the skin ulcer. Consequently, we sought consultation with the infectious disease service for appropriate antibiotic therapy and assessment of the risk of mastoid bone osteomyelitis. Fortunately, the MRI of the mastoid bone returned normal. Intravenous ciprofloxacin and oral metronidazole were initiated based on antibiogram results. Biopsy from the ulcer margin revealed perifollicular dermal abscess formation evolving into skin ulceration, pseudoepitheliomatous hyperplasia, dermal necrosis, extensive abscess formation, mixed acute and chronic inflammation, and inflammation of vascular walls, all strongly suggestive of pyoderma gangrenosum (Figure 2A–C). Screening for associated inflammatory bowel disease, rheumatologic disease, and hematological disease yielded no abnormalities. An abdominal and pelvic ultrasound was performed to detect any potential underlying malignancies, revealing a heterogeneous adnexal cyst on the left side. A subsequent pelvic MRI displayed a 101*39*79 mm multiloculated cystic mass with internal septa and a 26 mm mural nodule with enhancement in the left adnexa, highly suggestive of malignancy. The gynecologist recommended laparotomy for the excision of the pelvic lesion.

**FIGURE 3:**
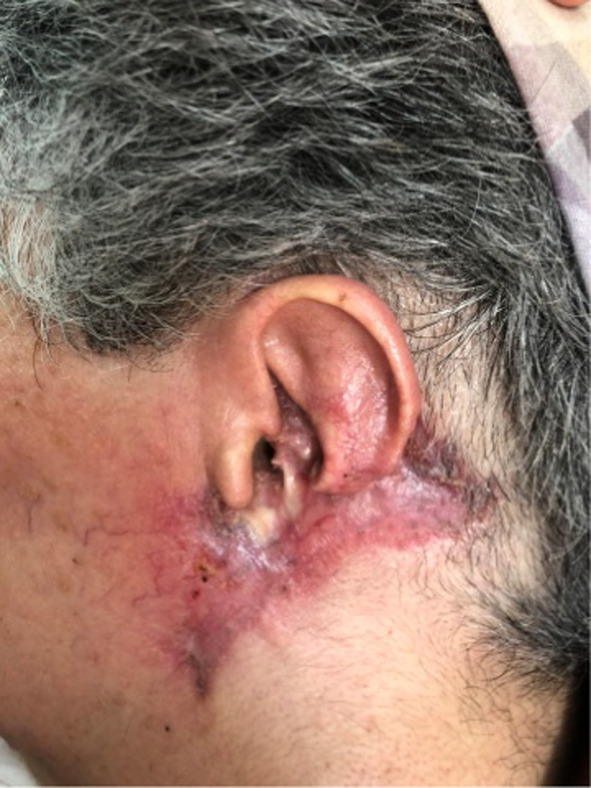
The ulcer healed completely after 4 months of follow‐up.

**FIGURE 4:**
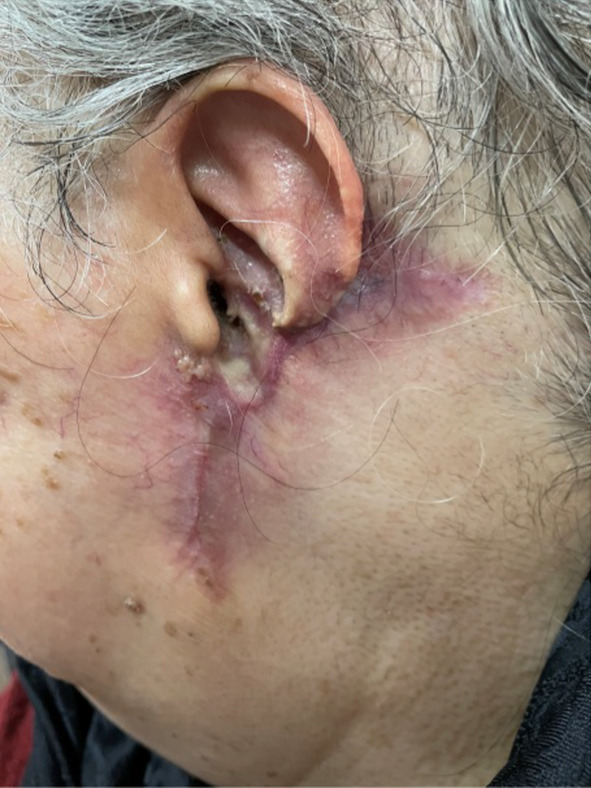
There is no recurrence and no new lesions after 12 months of follow‐up.

We initiated methylprednisolone pulse therapy (500 mg/day) for three consecutive days, followed by oral prednisolone 60 mg daily and oral cyclophosphamide 100 mg daily. After 2 weeks of treatment, ulcer's progression halted, and it gradually reduced in depth and width. The patient underwent laparotomy for tumor resection. Pathology of the mass revealed a malignant high‐grade epithelial neoplasm, positive for CKAE1/AE3, ER, PR, CK7, WT1, Desmin, and P53 and negative for CK20 in immunohistochemistry staining, suggestive of carcinosarcoma. Subsequently, she was referred to the oncology service for further cancer treatment, where the diagnosis of fallopian tube carcinosarcoma was confirmed. The oral corticosteroid was tapered and discontinued over 4 months, while cyclophosphamide was continued for 6 months under the oncologist's supervision. The ulcer healed completely, leaving a cribriform scar and no new lesions or recurrence were detected during 18 months of follow‐up (Figures 3 and 4).

## 3 DISCUSSION

In this report, we describe a case of facial PG associated with fallopian tube malignancy. Initially, Perry et al.^5^ described malignant pyoderma gangrenosum as a rare subtype of PG occurring on the face, neck, and upper trunk in 1968. However, Stuart Wernikoff et al.^6^ later considered malignant pyoderma gangrenosum and PG on the face and neck to be identical disorder in a 1987 review article.

The face is an uncommon site for PG, with only 7.8% of PG cases occurring on the head or neck in one of the largest case studies. The leg was the most common site of PG (77.7%), followed by the trunk (11.7%), peristomal region (8.7%), and upper extremities (8.7%).^7^ Betsy Ambooken et al.^8^ reported a 47‐year‐old woman with PG on the neck overlying the right parotid gland, which was treated effectively with dexamethasone pulse therapy.

As face is not a common location for PG, it is frequently misdiagnosed. Eran Shavit et al.^9^ reported a series of 5 patients with superficial granulomatous pyoderma gangrenosum on the face. In two of the cases, underlying disorders were detected, including ulcerative colitis and monoclonal gammopathy of undetermined significance.

The presentation of pyoderma gangrenosum‐like lesion before the diagnosis of GPA has been reported. Zarraga et al.^10^ reported a 50‐year‐old man with large necrotic ulcerations on the chest, upper arm, and parotidomasseteric regions which were very similar to PG in clinical manifestation; however, the elevated ANCA levels led the authors to the diagnosis of GPA. Similarly, Ekleva Jorgaqi et al. reported a 43‐year‐old male presented with multiple ulcerated lesions. Skin biopsies showed PG‐like features; nevertheless, the ANCA levels were elevated and 6 months later; the patient developed dyspnea with multiple focal opacities on the CT scan. The patient was diagnosed with GPA finally.^11^ However, in our case C‐ANCA and P‐ANCA levels, urine analysis and chest and paranasal sinuses CT‐scan all were normal and our patient had not presented any related systemic manifestation of GPA.

The association between PG and solid organ tumors (SM) has been the subject of previous research. A cross‐sectional investigation within a cohort study, consisting of 302 PG cases, is the largest study examining this relationship.^12^ In this study, no definitive connection between PG and SM was established. However, this contradicts earlier research findings. Two systematic reviews of prior studies have been conducted. Shah et al. conducted a comprehensive review of the literature, identifying 19 patients with concurrent PG and SM, with the majority (78.9%) having developed SM prior to PG. The most common malignancy accompanying PG was breast cancer. All patients reported achieving clinical remission, either through anti‐neoplastic or PG‐specific medication.^13^ Gupta et al. published a systematic review in 2019, analyzing 186 individual case reports of patients with PG and malignancy. In their report, 33 (17.7%) patients had SM, of which 71.0% had SM preceding PG.^14^ While the association between PG and malignancy remains debated, it cannot be disregarded. To our knowledge, the association between PG with carcinosarcoma of the fallopian tube has not been previously reported, making this case the first such report.

The treatment of pyoderma gangrenosum has been extensively studied, yet a standardized protocol applicable to all cases has not been universally accepted.^15^ Successful PG management requires not only medical therapy, but also wound care and appropriate dressing. Carlo Alberto Maronese et al. proposed an algorithm for PG management, categorizing it into mild and moderate‐to‐severe disease. Mild disease is defined by ulcers that are 3 cm or smaller in size, three or fewer than three in number, and involvement of less than 5% of the body surface area. Moderate‐to‐severe disease is characterized by ulcers larger than 3 cm, more than three in number, facial or genital involvement, and visualization of tendon, muscle, or bone. In mild cases, patients should receive topical or intralesional corticosteroids or topical calcineurin inhibitors initially. If no improvement is observed after 4–6 weeks, systemic therapy should be initiated. In moderate‐to‐severe cases, systemic treatment is the first‐line approach, involving systemic corticosteroids and/or cyclosporine. If no improvement is achieved within 4–6 weeks, biologics become the second‐line treatment option. In cases where biologics are contraindicated, immunosuppressive drugs such as methotrexate, mycophenolate mofetil, azathioprine, dapsone, colchicine, and thalidomide can be considered.^16^

This case highlights the importance of considering PG in the diagnosis of facial ulcers. Further investigation for underlying disorders is recommended, especially in unusual presentations. The association between PG and carcinosarcoma of the fallopian tube has not been previously reported. Treatment for PG should be individualized, and follow‐up is necessary to monitor for recurrence.

## AUTHOR CONTRIBUTIONS

**Ghazal Mardani:** Conceptualization; data curation; writing – original draft; writing – review and editing. **Mohammad Shahidi Dadras:** Visualization. **Fahimeh Abdollahimajd:** Conceptualization. **Toktam Safari Giv:** Data curation; writing – original draft. **Elnaz Pourgholi:** Data curation; writing – original draft. **Azadeh Rakhshan:** Investigation. **Fariba Ghalamkarpour:** Conceptualization; data curation; supervision; validation; writing – original draft; writing – review and editing.

## FUNDING INFORMATION

The authors received no financial support for the research, authorship, and/or publication of this article.

## CONFLICT OF INTEREST STATEMENT

All authors declare that they have no conflicts of interest.

## CONSENT

Patient provided written consent for the use of patient's photographs and related materials for publication.

## Data Availability

The data that support the findings of this study are available on request from the corresponding author. The data are not publicly available due to their containing information that could compromise the privacy of the patient.
